# Correction: The Links Between Parents’ Self-Stigma, Parental Burnout, Parental Competence, and Socio-Emotional Adjustment Among Parents of Children With Autism Spectrum Disorder

**DOI:** 10.1007/s10803-026-07475-1

**Published:** 2026-08-07

**Authors:** Yahya Aktu, Sabri Toğluk, Bilal Vural

**Affiliations:** 1 Department of Social Service and Counseling, Siirt University, Eruh, Siirt, Türkiye; 2 Department of Medical Services and Techniques, Siirt University, Siirt, Türkiye; 3 School of Health Sciences, Department of Child Development, Bitlis Eren University, Bitlis, Türkiye

**Correction to: Journal of Autism and Developmental Disorders**

**https://doi.org/10.1007/s10803-026-07405-1**

In this article the author’s name Sabri Toğluk was incorrectly written as Sabri Toğuk.

The original article has been corrected now.

